# The Prevalence of Exercise Addiction Symptoms in a Sample of National Level Elite Athletes

**DOI:** 10.3389/fspor.2021.635418

**Published:** 2021-06-10

**Authors:** Mia Beck Lichtenstein, Anna Katarina Melin, Attila Szabo, Lars Holm

**Affiliations:** ^1^Department of Clinical Research, Faculty of Health Sciences, University of Southern Denmark, Odense, Denmark; ^2^Center for Telepsychiatry, Mental Health Services in the Region of Southern Denmark, Odense, Denmark; ^3^Department of Sport Science, Linnaeus University, Växjö/Kalmar, Sweden; ^4^Institute of Health Promotion and Sport Sciences, ELTE Eötvös Loránd University, Budapest, Hungary; ^5^Department of Research, Team Danmark, Broendby, Denmark

**Keywords:** exercise addiction inventory, elite athletes, prevalence, validation, high volume exercise

## Abstract

Exaggerated exercise volumes, lack of control, withdrawal symptoms and conflicts with family and friends are core symptoms of exercise addiction. The condition can lead to health problems and social isolation because exercise is given the highest priority in any situation. The prevalence of the risk of exercise addiction has mostly been assessed in leisure time exercisers such as runners, fitness attendees and cyclists. The prevalence proportion ranges from 3 to 42% depending on the type of sport and the assessment tool. The proportion is greater among elite athletes, and increases with the level of competition. This study's primary aim was to assess the prevalence of exercise addiction among elite athletes competing at national level and its secondary aim was to evaluate the psychometric properties of the Exercise Addition Inventory (EAI) in elite sports. Participants (*n* = 417) from 15 sports disciplines and with 51% women completed an online survey. Results showed that 7.6% were at risk of exercise addiction. This group was younger, exhibited tendency to exercise despite pain and injury, felt guilty if not exercising enough, and reported substantial eating disorder symptoms. The reliability and validity of the EAI was good suggesting that the scale is appropriate for measuring the risk of exercise addiction in elite athletes.

## Introduction

Regular physical activity and planned exercise training are important in the maintenance of health and disease prevention in today's highly sedentary societies. Apart from leisure exercise, sports at various competition levels, also fulfill this purpose. However, sports can be highly demanding due to their competitive nature and high training demands, and athletes challenge their limits by often exhibiting obsessive, compulsive relationship with their sport (Cromer et al., 2017). The great pressure on national and international level athletes, may impose lack of control of their athletic situation and symptoms of exercise addiction (Çetin et al., 2020).

### Exercise Addiction

Addiction to exercise can be categorized as a behavioral addiction recognized by a behavior that is getting out of control (Szabo, 2010). Using exercise to regulate emotions, increasing exercise amounts to achieve a “high,” having conflicts with the family and withdrawal symptoms are core symptoms of exercise addiction (Brown, 1997).

Compulsive exercise seems to be associated with health problems, anxiety, depression and eating disorders (Weinstein et al., 2015, Lichtenstein et al., 2017b; Nogueira et al., 2018). Elite athletes with high risk of addiction may experience a negative impact on performance (Çetin et al., 2020).

Being an elite athlete (i.e., performing at the highest international level) case studies report high training volumes. A female skier conducted on average 940 h a year across a 5-year successful period (Solli et al., 2017) and a male biathlete up to 700 h per year (Schmitt et al., 2020). Thus, confusion in the distinction between commitment and addiction is obvious.

But the training amount in itself is not the main issue of exercise addiction. Well-planned periodic training patterns with days of rest in combination with a harmonious passion for sport is different from a constant need for increase of training load, lack of recovery, and an obsessive engagement in training (Lichtenstein et al., 2020). However, to date, few studies have addressed the symptoms of exercise addiction in elite athletes despite the over 1,000 publications in the field (Szabo and Kovacsik, 2019).

Exercise addiction *per se* cannot be diagnosed, because there are no clinical criteria for diagnosis. Indeed at this exercise addiction is not included in the latest edition of the “Diagnostic and Statistical Manual of Mental Disorders” (DSM-5; American Psychiatric Association, 2013). Therefore, studies on exercise addiction assess the *risk* of the dysfunction by using questionnaires. One of the commonly accepted questionnaires is the Exercise Addiction Inventory (EAI; Terry et al., 2004), described in more detail in the Methods section, which is based on the components model of addictions (Griffiths, 2005). This model entails six symptoms of addiction common to substance and behavioral addictions: salience, conflict, mood-modification, tolerance, withdrawal symptoms and relapse. The EAI was developed to gauge exercise addiction in leisure exercisers and has excellent psychometric properties in this population (Griffiths et al., 2015). It was also used with competitive athletes (De la Vega et al., 2016), who scored higher than leisure exercisers, but it has not been validated in elite athletes. A study aiming to investigate the prevalence of the risk of exercise addiction in elite athletes must ensure that the adopted instrument is valid and reliable.

### Prevalence of Exercise Addiction

The prevalence of indicated exercise addiction has mostly been studied in leisure time exercisers such as runners, cyclists, fitness center attendees and sport students. Research suggest that the prevalence is about 3% in the general exercising population (Mónok et al., 2012) but it can range from 3 to 42% (Szabo et al., 2015), depending on the type of exercise and the used assessment tool (Lichtenstein et al., 2017b; Nogueira et al., 2018; Di Lodovico et al., 2019; Marques et al., 2019). A recent literature review revealed that that there is a larger proportion of individuals at risk of exercise addiction among endurance athletes (14.2%), ball game players (10.4%), gym attendees (8.2%), strength disciplines (6.4%), compared to about 3.0% in other sports (Di Lodovico et al., 2019). Even higher rates (17%) have been reported in elite ultramarathon runners (Szabo et al., 2013), in Australian elite athletes (34.8%) (McNamara and McCabe, 2012).

A similar prevalence rate as reported in elite ultramarathon runners by Szabo (2018) was reported in Italian team-sport athletes in which 18.3% of 262 competitive athletes from nine different sports (basketball, futsal, football, handball, hockey, rugby, softball, volleyball, and water polo) were found to be at risk for exercise addiction (Costa et al., 2014). Based on these risk ratios stemming from the limited research with elite athletes, it appears that elite athletes are at greater risk of exercise addiction than recreational athletes. Some sports (e.g., endurance disciplines) may be at higher risk than sports where exercise volume is less important (e.g., football; Di Lodovico et al., 2019). The literature also need more studies exploring the prevalence of exercise addiction in adolescents since the development of addiction in early age is a risk factor of mental and social problems later in life (Marques et al., 2019). Therefore, a larger sample comprising different elite sport disciplines is needed to uncover the rate of the risk of exercise addiction across several types of sports and age groups.

### Eating Disorders

Exercise addiction may be part of the eating disorder pathology and therefore, often the eating disorder symptoms are considered separately in the assessment of exercise addiction (Cook and Luke, 2017). The reason for the commonality is that exaggerated exercise is also part of eating disorders in which it serves as a method of losing weight in addition to dieting. This type of exercise abuse is termed *secondary* exercise addiction (De Coverley Veale, 1987). In contrast, when exercise itself is the gist of the addiction the dysfunction is termed as *primary* exercise addiction (De Coverley Veale, 1987). Due to the great overlap between the risk of exercise addiction and eating disorders it is difficult to untangle the two. In elite athletes, weight focus is frequent and, consequently, it can be predicted that there is a close relationship between exercise addiction and eating disorder in this population. The association between exercise addiction and eating disorders in elite athletes has not previously been investigated.

### Aims

The aim of this cross-sectional study was to assess the prevalence of exercise addiction symptoms in elite athletes from 15 different types of sports and to characterize athletes with risk of addiction according to gender, age, body mass index (BMI), exercise volumes, recovery from exercise, injuries, feeling guilty if reducing exercise and eating disorder symptoms. We hypothesized that the risk of exercise addiction in Danish elite athletes would be similar to the reported prevalence in Italian and Spanish elite athletes (Szabo et al., 2013; Costa et al., 2014), and greater than earlier reported in recreational active people (Mónok et al., 2012; Szabo et al., 2013).

The secondary aim was to evaluate the psychometric properties of the Exercise Addiction Inventory in this sample of Danish elite athletes. Based on a cross-cultural evaluation of this instrument (Griffiths et al., 2015), we hypothesized that the Exercise Addiction Inventory would reveal good psychometric properties in a sample of Danish elite athletes.

## Materials and Methods

### Research Design

This study was a cross-sectional survey conducted in collaboration with Danish sport organizations that provided email-contact for Danish athletes training at the highest level of sport competition.

### Ethics

The research was conducted according to General Data Protection Regulation (GDPR), and the Regional Committees on Health Research Ethics for Southern Denmark confirmed that the anonymous questionnaire study did not need ethical approval. All participants (or parents for participants aged 15–17 years) gave written consent at the online survey to participate in the study. Participation was voluntary and anonymous.

### Participants

A sample of 1,058 Danish male (58%) and female (42%) national team athletes at junior and senior level, including national team recruiting squads, were invited by email to participate in the study. Three reminders were sent by email to all athletes, and the data collection was performed in December 2019 to March 2020. Participants could win a gift in a lottery if they gave information about their name and e-mail. Gifts were: three sets of tracksuits with the Olympic Games 2021 logo, two tickets to an annual sport gallery show, three body composition scans by Dual energy X-ray Absorptiometry, and 10 cinema tickets.

A total of 417 completed the survey (40% of the invited participants). The age span was 15–47 years, and 51% were females.

Athletes from the following sports were included in the study:

1. Endurance/antigravity sport (40.4% of the sample): Middle- and long-distance running, cycling, orienteering running, triathlon, swimming and jumping disciplines (e.g. long jump).
2. Weight category sport (11.2% of the sample): Karate, wrestling, boxing, and lightweight rowing.
3. Aesthetic sport (7.9% of the sample): Sports dance, ice skating, and gymnastics.
4. Other sports (40.4% of the sample): Badminton, handball, football, throwing disciplines (e.g. javelin), heavy rowing, and other sport (*n* = 11).

### Measures

All data were collected from the online survey where respondents were asked to report symptoms of exercise addiction and eating disorders.

EAI was developed in 2004 (Terry et al., 2004) from a theoretical model of behavioral addictions (Brown, 1997). The EAI covers six core symptoms of addiction and it has one item for each of the symptoms:

1. Exercise is the most important thing in my life
2. I have conflicts with family or friends because I exercise so much
3. I use exercise to change my mood (e.g., to feel happier or forget about problems)
4. Over the last year, I have increased the amount of daily exercise that I do
5. If I don't exercise every day, I get restless, upset or sad
6. I have tried to reduce the amount of exercise I do but end up exercising as much as I did before

Each item is scored on a Likert-scale from 1 (strongly disagree) to 5 (strongly agree) and a total score of 24–30 indicates high risk of addiction. EAI has shown good psychometric properties across countries and in different leisure time sport samples (Griffiths et al., 2015). The EAI has never been validated in a sample of elite athletes though they may have a higher risk of addiction to exercise. The internal structure of the scale and the underlying construct may differ because elite athletes are expected to perceive exercise as the most important thing in their lives. Training and competition are their job, and they have to train on a daily basis to obtain their goals and “keep the job.”

The Danish version of the EAI (Lichtenstein et al., 2014a) was used to assess the risk of exercise addiction using the cut-off score ≥24 as suggested by Terry et al. (2004).

The SCOFF (Sick Control, One Stone (6,5 kg), Fat, Food) is a short screening tool containing five items covering five symptoms of anorexia or bulimia nervosa (Morgan et al., 1999). The questions are responded with “yes” or “no.” Having two or more positive answers indicates risk of eating disorder pathology.

1. Do you make yourself **S**ick (induce vomiting) because you feel uncomfortably full?
2. Do you worry you have lost **C**ontrol over how much you eat?
3. Have you recently lost more than One stone in a 3-month period?
4. Do you believe yourself to be **F**at when others say you are too thin?
5. Would you say that **F**ood dominates your life?

SCOFF is recommended for use in large population studies because it is easy to administer and to fill out. However, it is not useful to establish diagnoses as it can only indicate a risk of eating pathology that needs further assessment of severity and duration.

The Danish version of SCOFF (Lichtenstein et al., 2017a) was used to assess risk of eating disorder symptoms using the cut-off score ≥2 as suggested by Morgan et al. (1999).

Furthermore, the respondents were asked to report age, gender, body mass index (BMI), training amounts, recovery from training, injury history, the tendency to exercise despite pain and injury and feeling guilty if reducing exercise.

### Statistics

Data were analyzed using Statistical Package for the Social Sciences (SPSS) version 26. Descriptive methods were used to compile participant characteristics. ANOVA was used to compare the mean score of the variables, and Chi-square was for categorical data. The internal consistency of the EAI-Y was tested with the Cronbach's Alpha (α) coefficient that is a function of the number of items in the EAI-Y, the average covariance between item-pairs, and the variance of the total score. A Principal Component Analysis (PCA) was used to test whether data were consistent with the hypothesis that the EAI represents one underlying component (or factor). The PCA was chosen similar to the study of Szabo et al. (2013), and we also used only components with eigenvalues of at least = 1.0.

Pearson's logistic regression models were to test construct validity of EAI-Y and related excessive and obsessive exercise attitudes/consequences: weekly exercise amounts, exercise in spite of pain and injury, feelings of guilt when not exercising, and reduced recovery.

A p-level of 0.05 is reported as statistically significant and between 0.05 and 0.10 as a statistical tendency.

## Results

### Prevalence

The prevalence of high risk of exercise addiction was 7.6% (*n* = 31 out of 410 respondents) and the mean EAI score was 17.7 ± 4.1. The prevalence in relation to type of sport showed inconsistency and non-significant differences: Endurance/gravity: 5.5%, Weight Category: 13.0%, Aesthetics: 3.0% Other sports: 8.9% (*p* = 0.22).

The total sample of elite athletes had the highest scores on item 1 (salience), item 2 (conflicts), and item 5 (withdrawal symptoms). Table 1 illustrates the distribution of scores on the six items of the EAI.

**Table 1:** Distribution of scores on the Exercise Addiction Inventory (EAI).

	**Strongly disagree**	**Disagree**	**Neither agree/disagree**	**Agree**	**Strongly agree**
EAI 1 The most important thing in life	2.9%	15.1%	27.6%	40.0%	14.4%
EAI 2 Conflicts with family and friends	5.9%	7.3%	17.6%	48.0%	21.2%
EAI 3 Emotion regulation	29.5%	34.4%	20.2%	12.4%	3.4%
EAI 4 Increasing exercise amounts	20.0%	35.1%	33.9%	10.0%	1.0%
EAI 5 Withdrawal symptoms e.g. restless, sad	8.5%	15.4%	22.4%	37.8%	15.9%
EAI 6 Loss of control	17.3%	35.4%	30.2%	12.9%	4.1%

### Characteristics of Athletes at Risk of Exercise Addictions

Table 2 shows the comparison of the high-risk group and the low-risk group of exercise addiction. Athletes with risk of exercise addiction had an equal distribution between genders. The mean age was lower among athletes with high risk of addiction with the highest risk in the youngest athletes (15–19 years). There was no difference in BMI or training volume between the groups, while the number of acute and overuse injuries during the last year tended to be higher in the high-risk group.

**Table 2:** Characteristic of athletes with risk of exercise addiction.

	**Risk of** ** exercise** ** addiction**	**No risk of** ** exercise** ** addiction**	**Significance**
Gender female/male	8.1%/7.0%	91.9%/93%	0.68
Age mean	18.5 (*SD* = 3.1)	20.1 (4.8)	0.061
15–19 years	8.8%	91.2%	0.45
20–24 years	6.3%	93.8%	
25–50 years	4.6%	95.4%	
BMI	21.3 (*SD* = 2.3)	21.8 (*SD* = 2.2)	0.26
Exercise volume/week	15.5 h/week (*SD* = 4.5)	15.8 h/week (*SD* = 6.3)	0.79
Overuse injuries last year	1.46 (*SD* = 1.35)	1.1 (*SD* = 1.02)	0.075
Recovery after exercise			0.12
After every session	48.8%	36.9%	
After many sessions	29.0%	49.9%	
After some session	22.6%	12.7%	
Rarely or never	0.0%	0.5%	
Exercise despite pain and injury			<0.001
Strongly agree	32.3%	4.7%	
Agree	32.3%	26.9%	
Neither agree/disagree	22.6%	21.9%	
Disagree	6.5%	26.4%	
Strongly disagree	6.5%	20.1%	
Feeling guilt			<0.001
Strongly agree	48.4%	7.1%	
Agree	16.1%	27.4%	
Neither agree/disagree	19.4%	21.9%	
Disagree	6.5%	28.5%	
Strongly disagree	9.7%	15.0%	
Eating disorder risk	15.5%	5.9%	0.005

Among athletes with risk of addiction almost 2/3 agreed “to exercise despite pain and injury,” while another 20% reported that they neither agreed nor disagreed. The same results were found regarding the item “feeling guilty about not exercising enough.”

Recovery after training seemed to be good in both groups. The risk of eating disorder symptoms was higher in the addiction group.

### Reliability of the Exercise Addiction Inventory

The internal consistency of the six items in EAI was evaluated with Cronbach's Alpha finding a coefficient of 0.72.

The PCA showed factor loadings on all six items above 0.58 supporting the inclusion of each EAI-item in the model. Item three (emotion regulation) had the lowest loading at the first factor. The factor loadings on the six items (see Table 1) were: 0.61, 0.66, 0.58, 0.67, 0.66, and 0.68, respectively.

**Figure 1:**
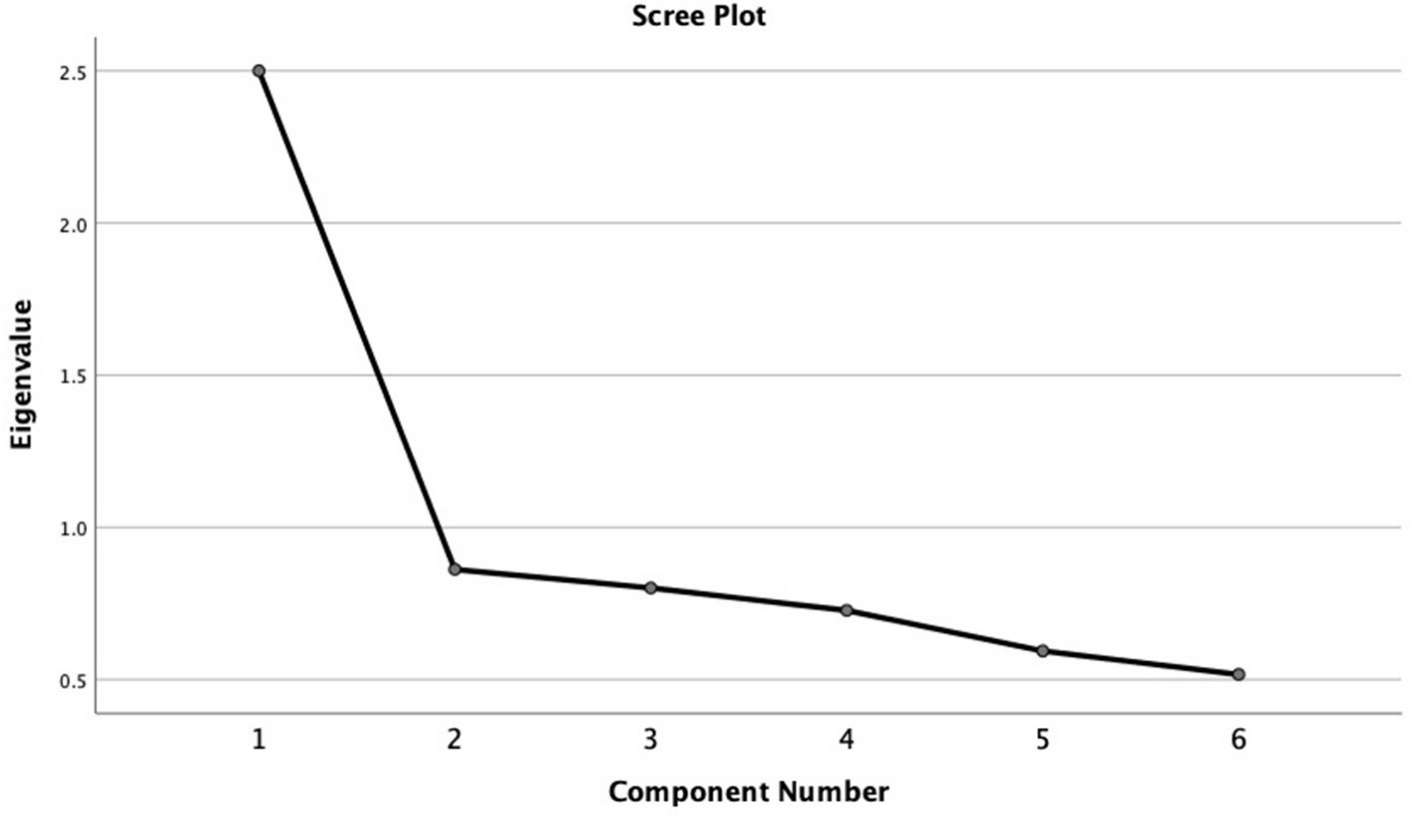
Scree plot of the factor structure of the Exercise Addiction Inventory.

The first factor explained 41.7% of the variance with an eigenvalue of 2.5. Adding a second factor contributed with only 14.4%, indicating a single factor structure of the EAI (see scree plot in Figure 1).

### Construct Validity of the Exercise Addiction Inventory

The construct validity was evaluated by regression models showing that higher scores on exercise addiction correlated positively with higher scores on “exercise despite pain and injury” *r* = 0.32 (*p* < 0.001) and “feeling guilty not exercising enough” *r* = 0.43 (*p* <0.001).

There was no association between exercise addiction scores and training volume *r* = 0.04 (*p* = 0.48).

## Discussion

### Prevalence of Exercise Addiction

Our main finding was that 7.6% of elite athletes representing 15 different sport disciplines were at risk of exercise addiction, that was also associated with an increased risk of eating disorders. The connection between the risk of exercises addiction and overexercising for the sake of diet and weight control is difficult to untangle. The risk of exercise addiction occurs three and a half times more frequently, as a co-morbidity in people suffering of some sort of eating disorder in contrast to individuals without an eating disorder (Trott et al., 2020). Exaggerated exercise in the latter group is known as *primary* exercise addiction, in which exercise fulfillment is the reward itself, while in various eating disorders exercise is only an additional means (above diet, purging, etc.) in weight control and, therefore, it is referred to as *secondary* exercise addiction (De Coverley Veale, 1987). However, since exercise is a crucial means of weight loss for many suffering from eating disorders, these individuals are likely to score high on both exercise addiction and eating disorders measures. In our study with athletes, the connection generally might be related to weight control imposed by athletic requirements rather than morbid eating disorder, which would incapacitate the optimal athletic performance. Eating disorder symptoms are often present in recreational addicted exercisers and may be the primary problem (Trott et al., 2020), but this association has not yet been explored in elite athletes. Here, we found that 16% of athletes with high risk of addiction had symptoms of an eating disorder compared to 6% of low-risk athletes. Thus, based on these findings we recommend screening elite athletes with exercise addiction for eating disorder pathology and vice versa. However, future studies, should untangle the extent to which eating disorder symptoms in athletes are related to weight concern associated with the athletic career and dysfunctional eating habits.

Our findings agree with previous research exploring the prevalence of exercise addiction in different sub-samples, mostly concerning fitness exercisers, runners, sports students, and other non-elite populations (Lichtenstein et al., 2017b; Di Lodovico et al., 2019). In the context of elite athletes, our findings are lower than that reported by the few other studies in the area (McNamara and McCabe, 2012; Szabo et al., 2013; Costa et al., 2014). Still they agree, with the prevalence rate of 7.1% obtained in another earlier Danish study with football players (Lichtenstein et al., 2014b). The discrepancy with the other studies can be ascribe to several factors, including cross-cultural differences (Griffiths et al., 2015), different instruments used (McNamara and McCabe, 2012; Costa et al., 2014), and factors that were not investigated in these studies, such as, athletic performance (Çetin et al., 2020).

We found that the prevalence of exercise addiction symptoms was numerically the highest in weight disciplines (13.0%) and in other sports, including ball games (8.9%), and lowest in gravity/endurance sport (5.5%) and aesthetic sports (3.0%). None of these prevalence proportions were, however, statistically different.

This finding is not matching the results of the review by Di Lodovico et al. (2019) who found that endurance disciplines had the highest risk of addiction (14.2%) compared to mixed disciplines (10.4%) and fitness (8.2%), while power disciplines have a lower prevalence (6.4%). The different findings, again like with prevalence rates, might be due to factors related to culture, level of competition, training intensity, training history, and many others that were not generally assessed in studies examining exercise addiction. For example, Szabo et al. (2013) found that team sports scored higher on exercise addiction compared to individual sports, while in an earlier work we found no differences between team and individual exercisers (Lichtenstein et al., 2014b). This non-significant tendency was the same in our current study where “other sport” (including handball and football) had a higher prevalence of exercise addiction symptoms than endurance sports, including running, cycling, swimming, and other individual disciplines.

The differences in the rate of the risk of exercise addiction within and between sports makes it impossible to establish *normative* values. Further, it cannot be predicted how many of those in the “at risk” group will ever become addicted to exercise in a dysfunctional manner. Still, our study lends support to the bulk of the literature that, in general, showing that the prevalence of the risk of exercise addiction is greater in elite athletes than the rate (3–5%) reported for recreational exercisers (Mónok et al., 2012). The reason behind this repeatedly demonstrated difference is subject to future empirical research, while it is not impossible that conceptual issues associated with the assessment of the risk of exercise addiction may yield false figures in elite athletes (Szabo et al., 2015; Szabo, 2018). In this context, it should be remembered that the original EAI (Terry et al., 2004) was developed for recreational exercisers, even though its psychometric properties appear to be good in elite athletes too, as revealed in the present work.

### Characteristics of the at Risk of Addiction Group

The addiction group was characterized by lower age, a high risk of exercise despite pain and injury, feelings of guilt regarding exercising enough, and more eating disorder symptoms. Our results support the findings derived from empirical and review studies (McNamara and McCabe, 2012; Lichtenstein and Jensen, 2016; Nogueira et al., 2018) that engagement in exercise despite physical injury or illness is associated with exercise addiction.

This characteristic of the risk group shows that exercise addiction in elite athletes may compose a vulnerable group of athletes. Especially young athletes in the age 15–19 years had a higher risk of addiction (8.8%) in the current study. A review by Marques et al. (2019) investigating prevalence of exercise addiction in different samples concluded that adolescence is a period of greater risk for developing addiction and reported a similar prevalence of 9% in youth.

The exercise amount was equal in both our groups, indicating that athletes can perform high volume exercise (15–16 h a week) without suffering from addiction symptoms. A study by Szabo et al. (2013) also found that in elite athletes, there was no difference between addicted and non-addicted according to exercise volume, but in recreational exercisers, higher addiction scores are related to higher weekly exercise volume (Terry et al., 2004; Szabo et al., 2019).

This finding confirms that the nature of exercise addiction is not just large exercise amounts but rather the relationship to exercise (does it control your life, is the family concerned, do you experience withdrawal symptoms, etc.). However, all elite athletes may perform high training volumes, thus making comparisons between groups difficult.

### Psychometric Evaluation

The current Danish study was the first to conduct a psychometric examination of the EAI in a sample of national level elite athletes, and we conclude that the reliability of the scale is good and comparable to the results obtained with recreational exercisers. The internal reliability of the scale was good (Cronbach's *a* = 0.72), which is in accord with the range reported in a cross-cultural evaluation of the EAI (Griffiths et al., 2015), and suggests that all six EAI-items are useful assessing the risk of exercise addiction in elite athletes.

A study from Spain (Szabo et al., 2013) also evaluated the reliability and factor structure of EAI and found results very close to our research in terms of a Cronbach's Alpha coefficient of 0.71 and 42.0% of the variance explained by the first factor with factor loadings of minimum 0.4 observed for each item. This study used a mixed sports sample comprising two university student samples (*n* = 57 + 90) and elite athletes (*n* = 90).

The construct validity was acceptable with a significant association between high EAI-scores and high scores on attitudes expected to be associated with addiction behavior (exercise despite negative consequences and distress related to exercise patterns). The same relationship has been seen in recreational exercisers (Lichtenstein et al., 2018a,b), indicating that the construct of exercise addiction may be the same regardless the competitive level.

Contrary to this hypothesis is the finding of De la Vega et al. (2016), who suggest that exercise addiction is not the same phenomenon in competitive as in non-competitive athletes. This view is echoed by other too (Szabo et al., 2015; Szabo, 2018). These scholars believe that competitive athletes may interpret the EAI items as signs of passion and dedication, reflecting a positive perspective on the addiction symptoms. The authors conclude that EAI and other instruments used to assess exercise addiction in leisure exercisers may not be useful in competitive athletes.

The present study does not support this conclusion. Based on our psychometric evaluation of EAI in a large sample of elite athletes, we suggest that EAI is applicable in detecting the risk exercise addiction in elite athletes. However, more extensive validations covering different sports in different languages are needed to conclude that EAI measures the same construct as in non-elite samples. We also suggest an examination of the *perceived meaning* of the items on EAI for elite athletes and recreational exercisers, which could shed light on the differences between the two populations observed in the prevalence proportions.

### Strengths and Limitation

A strength of this cross-sectional study is that it examined a hard-to-approach sample of national elite athletes from 15 different sports. Indeed, this is the largest sample ever of elite athletes ever examined in the context of the risk of exercise addiction. Another noteworthy strength is that the current study included young (non-adult) athletes, which again are difficult to study due to ethical constrains and parental consent. Moreover, the study results revealed that the prevalence of the risk of exercise addiction appears to be the greatest in this group. Another strength of the work is that it has evaluated the tool with which the reported data were obtained. This control for the instrument was necessary, because although several validated versions of the EAI exist, the tool was not tested in a purely high level of athletes.

The study has several limitations too. First, the volunteer and anonymous sample, along with online responses, which all raise issues of control over the study, is the main limitation in the current work. Another limitation of the current study, out of the researchers' control, is the low response rate (40% of athletes) which could bias the prevalence estimates. Self-selected athletes may be those with a personal interest in the topic, or it may be those who are not affected, leading to either too high or too low prevalence rates, thus they may not be representative of the general athletic population. The lack of a non-athlete control group, or athletes competing at lower levels, is another limitation of the work. Finally, given that different tools may yield different prevalence rates of exercise addiction (Mónok et al., 2012), the lack of use of another exercise addiction measure is another limitation of the study. Thus, the validity findings should be interpreted with caution.

## Conclusions

In conclusion, this study found that 7.6% of elite athletes representing 15 different sport disciplines were at risk of exercise addiction. The reliability and validity of the scale revealing this finding appears to be good in elite athletes. Accordingly, the EAI is useful in detecting the risk of exercise addiction in national level elite athletes addiction. Athletes in the high risk of exercise addiction group were the youngest, exhibited a high risk of exercise-involvement despite pain and injury, reported feelings of guilt if the planned exercise volume is not accomplished reported poorer recovery after exercise, and, perhaps, most importantly revealed more eating disorder symptoms than athletes who were not at risk of exercise addiction.

## Data Availability Statement

The raw data supporting the conclusions of this article will be made available by the authors, without undue reservation.

## Ethics Statement

Ethical review and approval was not required for the study on human participants in accordance with the local legislation and institutional requirements. Written informed consent to participate in this study was provided.

## Author Contributions

The research design was developed by ML and AM. The data-collection was conducted by ML. All authors have contributed to data-analyses and writing the manuscript and have approved the submitted version.

## Conflict of Interest

The authors declare that the research was conducted in the absence of any commercial or financial relationships that could be construed as a potential conflict of interest.
